# Recent Progress in Designing Stable Composite Lithium Anodes with Improved Wettability

**DOI:** 10.1002/advs.202002212

**Published:** 2020-10-11

**Authors:** Zi‐Jian Zheng, Huan Ye, Zai‐Ping Guo

**Affiliations:** ^1^ Hubei Collaborative Innovation Center for Advanced Organic Chemical Materials Key Laboratory for the Green Preparation and Application of Functional Materials Ministry of Education Hubei Key Laboratory of Polymer Materials School of Materials Science and Engineering Hubei University Wuhan 430062 P. R. China; ^2^ College of Science Huazhong Agricultural University Wuhan 430070 P. R. China; ^3^ School of Mechanical, Materials, Mechatronic, and Biomedical Engineering Faculty of Engineering and Information Sciences University of Wollongong Wollongong NSW 2522 Australia

**Keywords:** high stability, lithium anodes, lithium metal batteries, long‐term life, wettability

## Abstract

Lithium (Li) is a promising battery anode because of its high theoretical capacity and low reduction potential, but safety hazards that arise from its continuous dendrite growth and huge volume changes limit its practical applications. Li can be hosted in a framework material to address these key issues, but methods to encage Li inside scaffolds remain challenging. The melt infusion of molten Li into substrates has attracted enormous attention in both academia and industry because it provides an industrially adoptable technology capable of fabricating composite Li anodes. In this review, the wetting mechanism driving the spread of liquefied Li toward a substrate is discussed. Following this, various strategies are proposed to engineer stable Li metal composite anodes that are suitable for liquid and solid‐state electrolytes. A general conclusion and a perspective on the current limitations and possible future research directions for constructing composite Li anodes for high‐energy lithium metal batteries are presented.

## Graphical abstract

Infusing molten Li into framework structured substrates helps address the safety hazards of Li metal, but strategies to entrap Li metal inside the scaffolds presents a major challenge. This review focuses on the recent achievements in constructing stable Li metal composite anodes by the thermal infusion method with control of the wetting mechanism.

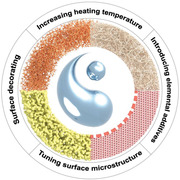

## 1 Introduction

Li metal anodes have attracted enormous attention because Li possesses a high theoretical capacity (3860 mA h g^−1^), low reduction potential (−3.04 V vs standard hydrogen electrode), and a low density (0.53 g cm^−3^), which holds promise for energy storage systems with high energy density.^[^ ^1^, ^2^, ^3^, ^4^, ^5^ ^]^ Li‐LMO cells (where LMO refers to lithium transition‐metal oxide) have a high theoretical gravimetric energy density of 440 Wh kg^−1^, which is double that of cells based on graphite anodes (250 Wh kg^−1^). Rechargeable lithium metal batteries (LMBs) assembled using a sulfur or oxygen cathode have delivered energy densities of 650 and 950 Wh kg^−1^, respectively.^[^ ^6^ ^]^ Despite the significant improvements in energy density, the commercialization of advanced LMBs is severely limited due to their poor safety related to Li dendrite growth and the significant volume changes of “hostless” Li.^[^ ^7^, ^8^, ^9^, ^10^ ^]^

To overcome these obstacles, progress has been made through the optimization of organic electrolytes,^[^ ^11^, ^12^, ^13^ ^]^ the creation of protective interface layers,^[^ ^14^, ^15^, ^16^ ^]^ solid electrolyte exploration,^[^ ^17^, ^18^, ^19^ ^]^ and 3D‐designed hosts for Li metal.^[^ ^20^, ^21^, ^22^, ^23^, ^24^ ^]^ 3D Li hosts effectively homogenize the Li‐ion flux, regulate uniform Li nucleation and growth, stabilize Li/electrolyte interfaces, and prevent dimensional variation of the electrode due to their high specific surface area and porous structures.^[^ ^25^, ^26^, ^27^ ^]^ Nevertheless, there are great technical challenges associated with encapsulating metallic Li inside 3D scaffolds. Electrochemical deposition is commonly used to predeposit metallic Li on 3D substrates, followed by the transfer and recombination of treated Li anodes through cell disassembly/reassembly.^[^ ^28^, ^29^, ^30^ ^]^ Predeposition is time‐consuming and expensive, however. Recent attempts have been made to press 3D hosts into metallic Li to prepare 3D host/Li composites,^[^ ^31^, ^32^, ^33^ ^]^ but these typically result in poor interfacial contact between the 3D host and the Li metal.

It is desirable to develop a versatile and facile approach for entrapping Li inside porous scaffolds to create 3D composite Li anodes. Li metal has a low melting point of 180 °C, and thus, thermal infusion strategies in which molten Li is infused into a “lithiophilic” matrix have been developed,^[^ ^34^, ^35^, ^36^ ^]^ but the underlying wetting mechanism is not fully understood. Understanding the underlying wetting mechanism, and thus how to tune the microstructure/composition on substrate surfaces or Li droplet characteristics, provides a guide for designing stable Li metal composite anodes. Compared with Li anodes prepared through predeposition or physical compression strategies, Li metal composite anodes prepared via thermal infusion methods have dense Li deposition, which ensures a high volumetric energy density. This method also provides intimate interfacial contact between Li anodes/electrolytes (particularly solid‐state electrolytes), enabling low interfacial impedance. It has a high universality toward various substrates, especially nonconductive substrates, and is an industrially adoptable technology for large‐scale preparation.

This review aims to provide guidance for the construction of 3D composite Li anodes by first reviewing the two main factors that influence wettability: surface tension and Laplace pressure. Then, recent progress in developing 3D composite Li anodes is summarized, and the underlying mechanisms are also analyzed. Likewise, the challenges for constructing composite Li anodes via thermal infusion methods that are suitable for liquid and solid‐state electrolytes are also compared. A general comparison and a brief perspective on the current strategies and possible future research directions for constructing composite Li anodes for high‐energy LMBs are also provided.

## 2 Wettability Mechanism

### 2.1 Surface Tension

**Figure 1:**
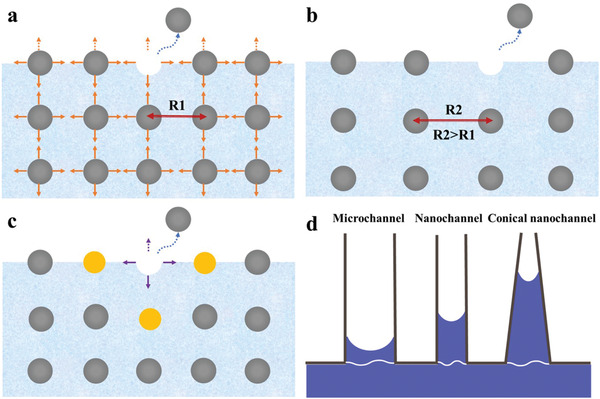
a) Definition of surface energy. b) The effect of temperature on the surface tension. c) The effect of heteroatomic doping on the surface tension. The yellow balls refer to the heteroatoms. d) Schematic representation of capillary force at different scales.

Wettability is a key property of material surfaces, and it plays a crucial role in preparing composite Li anodes via melt infusion strategies. It is commonly thought that this is mainly governed by the surface composition, which determines the surface free energy. Surface energy is defined as the strength of the cohesive forces per unit area that are required to pull interior particles into a surface (**Figure** **1**a). Many metals have high surface energies (about ten times that of water) because of their strong internal metal bonds.^[^ ^37^ ^]^ For pure liquids, the surface energy is numerically and dimensionally equal to the equivalent surface tension.^[^ ^38^ ^]^ The high surface tension of liquid metals drives them to contract into spherical structures which makes it difficult for them to spread onto substrates. Revealing the mechanism responsible for lowering surface tension can provide a biomimetic approach to the design of composite Li anodes.

Since surface tension is proportional to the mutual effects of the interior neighboring atoms, weakening the metallic bonding enhances the surface wettability. Most metals experience faster thermal motion at high temperatures than at low temperatures, which expands the distance between neighboring atoms and weakens the interior interactions, facilitating the “hopping” of metal atoms from the interior to the surface (Figure 1b). Thus, elevating the temperature appears to be a promising approach for decreasing the surface tension to improve the wettability of molten Li metal.^[^ ^39^ ^]^ The relationship between surface tension and temperature is expressed by Equation (1)

(1) $$\begin{array}{c} \gamma_{T}=\gamma_{m}+\frac{d\gamma }{dT}\left(T-T_{m}\right) \end{array}$$

where *γ*_T_ denotes the surface tension at temperature *T*, *γ*_m_ is the surface tension at the melting point, *T*_m_ refers to the melting point, *T* denotes the heating temperature, and d*γ*/d*T* < 0.

Heteroatomic doping is another feasible strategy to regulate the wettability of molten metals.^[^ ^40^ ^]^ Because the bonding force between the heteroatoms and Li is smaller than that of Li–Li, introducing a trace amount of elemental additives to pure metals to form alloys or compounds can reduce the interior atomic interactions to decrease the surface tension (Figure 1c). From a thermodynamic perspective, the Gibbs free energy (∆*G*), which governs the reactions between Li and elemental additives, determines the spontaneity of reactions. That is, a negative ∆*G* for the reaction between Li and elemental additives drives the spread of molten metals and improves the wettability.^[^ ^41^ ^]^ According to a previous study,^[^ ^42^, ^43^ ^]^ the wettability can be modeled by the contact angle, as in Equation (2)

(2) $$\begin{array}{c} \cos\theta^{1}=\cos\theta^{0}-\frac{\Delta \gamma_{\mathrm{sl}}}{\gamma_{\mathrm{gl}}}-\frac{\Delta G}{\gamma_{gl}} \end{array}$$

where *γ*_gl_ is the gas–liquid interfacial energy, ∆*γ*_sl_ refers to the change in the solid‐liquid interfacial energy due to changes in the physicochemical nature of the secondary‐phase Li‐based compounds formed at the interface, *θ* ^0^ denotes the contact angle without reaction, *θ* ^1^ is the contact angle after a reaction, and ∆*G* is the Gibbs free energy of the reaction. According to Equation (2), decreasing *γ*_sl_ (∆*γ*_sl_ < 0) and a negative ∆*G* released by the reaction can decrease *θ* ^1^ and increase the wettability. A more negative ∆*G*, a larger cos*θ* ^1^, and a smaller contact angle (*θ* ^1^) can all improve the wettability.

The spreading of molten metals is greatly determined by their surface tension, which can be decreased by increasing the external temperature or by decreasing interior atomic interactions by introducing elemental additives to form alloys or compounds.

### 2.2 Laplace Pressure

The Laplace pressure (Δ*P*), shown in Equation (3), arises from the surface microstructure of materials and acts as another driving force to alter the wettability of molten Li

(3) $$\begin{array}{c} \Delta P=\frac{2\sigma }{r} \end{array}$$

where *r* is the radius of the capillary and *σ* is the surface tension of the liquid Li. According to Equation (3), the smaller the radius *r*, the greater the Laplace pressure and the higher the liquid level will be, allowing nanochannels to produce larger capillary pressures than their microchannel counterparts (Figure 1d). Compared with common vertical nanochannels, conical nanochannels are regarded as the most ideal channel alternatives, because their conical geometry gives rise to a difference in Laplace pressure (Figure 1d). The Laplace pressure on the high‐curvature portion of the conical capillary is greater than on the low‐curvature portion, and the resultant nonequilibrium Laplace pressure drives Li droplets to move from the host's surface to its interior (Figure 1d). The process is similar to how trees transport water from their roots to their leaves using differences in Laplace pressure. Therefore, the design of host materials with nanochannels or surface microstructures has been used to tune the wettability of molten Li.

## 3 Composite Li Anodes in Liquid–Electrolyte Batteries

The wettability of Li refers to the spreading of liquid Li droplets with low surface tension over a substrate above its melting point (180.5 °C). Li droplets with high surface tension tend to ball‐up to avoid contact with the substrate. As stated, elevating the temperature and introducing elemental additives into Li are two effective ways to decrease the surface tension and improve the wettability. Here, we present wetting‐mechanism methods for fabricating composite Li anodes that are suitable for liquid electrolytes.

### 3.1 Li Droplet Characteristics

**Figure 2:**
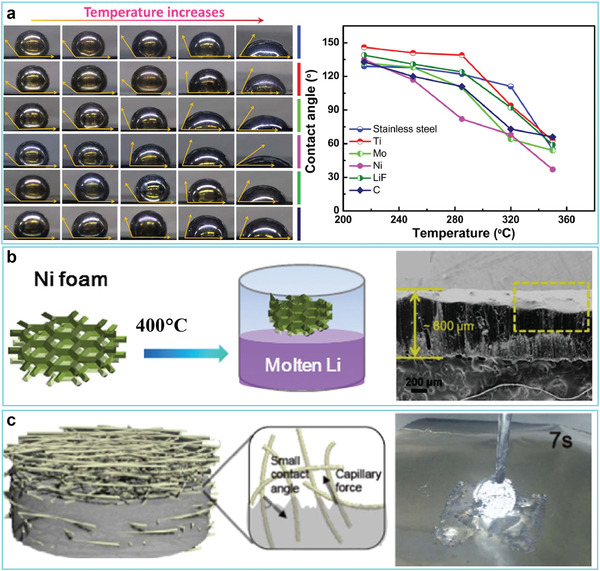
a) The effect of temperature on wetting by liquid Li. Reproduced with permission.^[^ ^44^ ^]^ Copyright 2018, Elsevier. b) Schematic representation of the preparation of Li–Ni composite anode. Reproduced with permission.^[^ ^45^ ^]^ Copyright 2017, Wiley‐VCH. c) Schematic illustrating of the preparation of Li–Cu@Ni nanocomposite anode. Reproduced with permission.^[^ ^46^ ^]^ Copyright 2017, Elsevier.

Increasing the temperature can weaken the interatomic forces of Li. Thus, increasing the temperature, decreasing the surface tension, and thus improving the wettability of molten Li is a convenient method for preparing 3D host/Li composites. Within this context, Cui and co‐workers explored the wettability of liquid Li on six types of superlithiophobic substrates, including stainless steel, titanium, molybdenum, nickel, lithium fluoride, and carbon foil, at various heating temperatures.^[^ ^44^ ^]^ The results showed that the contact angle between liquid Li and the substrates decreased as the temperature increased. All six substrates possessed a similar lithiophobicity at 215 °C with contact angles larger than 120° (**Figure** **2**a). When the temperature increased to 320 °C, the contact angles of all five substrates, except for stainless steel, decreased to 90°, demonstrating a transition from lithiophobicity to lithiophilicity. At 350 °C, the contact angles of all the substrates decreased to <90°, and the contact angle for Ni foil even decreased to about 40°, exhibiting complete lithiophilicity (Figure 2a). Of particular interest, Zhang and co‐workers realized the infusion of molten Li into superlithiophobic Ni foam at 400 °C (Figure 2b).^[^ ^45^ ^]^ Due to confinement of metallic Li by Ni skeleton, the thus‐formed Li–Ni composite electrode exhibited a small volume change (about 3.1%) in a carbonate‐based electrolyte. Likewise, Yu and co‐workers^[^ ^46^ ^]^ designed a 3D porous Cu@Ni nanowire scaffold to host Li (Figure 2c). Due to the low surface tension and strong capillary forces of the porous nanostructure at 400 °C, molten Li was easily taken up by the Cu@Ni nanowires (Figure 2c). Although these findings are significant, since they provide a simple and efficient strategy to construct composite Li anodes, the potential safety hazards originating from the high reactivity of Li and the low flash point of liquid solvents in the glove box restrict the use of high temperatures. Additionally, the higher energy consumption when using higher temperature means a higher cost to prepare 3D host/Li composites. More importantly, even higher temperatures may be needed for other substrates due to their intrinsic differences, which would further limit their practical applications.

**Figure 3:**
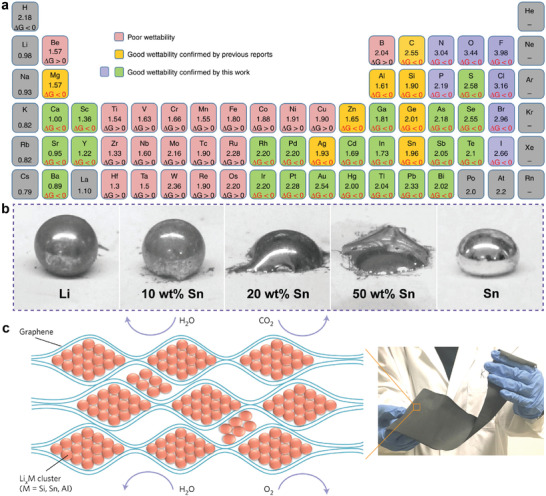
a) Δ*rG* of reactions between molten Li and possible elements or compounds in the periodic table at temperatures from 180 to 300 °C. Reproduced with permission.^[^ ^47^ ^]^ Copyright 2019, Springer Nature. b) Optical observations of the wettability of molten Li–Sn alloys with different ratios of Sn on alumina substrates. Reproduced with permission.^[^ ^48^ ^]^ Copyright 2018, Wiley‐VCH. c) Schematic representation of the current collector by sandwiching the reactive Li*_x_*M nanoparticles into graphene sheets. Reproduced with permission.^[^ ^58^ ^]^ Copyright 2017, Springer Nature.

In addition, alloying strategies have been developed to generate Li‐X (where X denotes different elemental additives) hybrids or coatings to lower the interior atomic binding forces of Li. This lowers the surface tension and facilitates the rapid spreading of molten Li. Additionally, the thus‐formed alloy phase can function as a framework to keep the volume of the Li metal anode stable and guarantee the long‐term cycling life of Li anode. Furthermore, the alloy phases offer Li nucleation and growth sites, and direct dendrite‐free Li deposition. From a thermodynamic viewpoint, a negative Δ*G* can be used to evaluate the spontaneity of an alloying reaction. In this context, Guo and co‐workers calculated Δ*rG* for reactions between molten Li and all known elements from 180 to 300 °C (**Figure** **3**a).^[^ ^47^ ^]^ The negative values of Δ*rG* for a specific reaction indicate improved wettability after alloying, which provides a comprehensive guide for tuning the wettability through elemental additives. Later, wettability experiments were performed to verify the improved wettability, and lower contact angles were obtained after the introduction of a series of elemental additives, including S (10 wt%), Sc (20 wt%), Mg (28 wt%), Te (40 wt%), Ga (40 wt%), Y (40 wt%), Ca (40 wt%), Se (50 wt%), Bi (50 wt%), Pb (50 wt%), Cd (50 wt%), Hg (50 wt%), Pd (50 wt%), Sr (50 wt%), In (50 wt%), and Ba (50 wt%), into molten Li. Following this work, Sn,^[^ ^48^ ^]^ Mg,^[^ ^49^ ^]^ In,^[^ ^47^ ^]^ C,^[^ ^50^ ^]^ and/or their compounds^[^ ^51^ ^]^ were investigated as dopants. For instance, Hu and co‐workers^[^ ^48^ ^]^ developed molten Li–Sn alloys (20–50 wt% Sn), which Li could rapidly wet (Figure 3b). The liquid Li–Sn alloys could be absorbed into garnet pellets within 10 s at 250 °C. Specifically, molten Li–Sn alloys exhibited good wettability on ceramics, metals, and polymer substrates, making them ideal for Li anode fabrication. Furthermore, Li‐rich Li–Mg alloys were easily spread onto the garnet substrate due to the decreased surface tension.^[^ ^49^ ^]^ Ca, Zn, and Al can react with Li to form lithium‐rich Li*_x_*M alloys which are strongly bridged with Li, boosting the nucleation and growth of metallic Li.^[^ ^52^, ^53^, ^54^ ^]^ Compared with pure Li, Li droplets containing C and C_3_N_4_ more rapidly adhered to the substrate.^[^ ^50^, ^51^ ^]^ Graphene has a high specific surface area, high electrical conductivity, and good mechanical strength, so it has been used as an efficient host to alleviate large volume expansion/contraction in order to maintain the stability of anodes.^[^ ^55^, ^56^, ^57^ ^]^ Cui and co‐workers^[^ ^58^ ^]^ developed self‐supporting Li*_x_*M (M = Si, Sn, or Al) alloy/graphene composite anodes using a prealloying reaction, followed by physical mixing (Figure 3c). The composite anode exhibited stability in ambient air, which enabled full‐cell assembly in ambient air. There is no doubt that alloy strategies are highly versatile and applicable to most substrates. Nevertheless, the rapid infusion is realized at the expense of increased elemental additive content in the liquid Li. The excessive introduction of additives may compromise the overall specific capacity of composite Li anodes and even the energy density of the full batteries. The heavy utilization of rare elements will also increase the costs of batteries. It is highly desirable to explore low‐cost elemental additives in trace amounts to balance the Li wettability and the specific capacity of composite Li anodes.

### 3.2 Substrate Characteristics

#### 3.2.1 Surface Micro/Nanostructures

Regulating the surface micro/nanostructures of current collectors is an effective method to boost the wettability of liquid Li. Materials with micro/nanochannels can produce large capillary pressures that drive liquid from the bottom to the top. Thus, current collectors designed with vertical micro/nanoscale channels or crevices have been employed as efficient hosts for molten Li.

Recently, Zhou and co‐workers reported that CoO nanofibers on Ni foam displayed good Li wettability.^[^ ^23^ ^]^ The accumulation of nanofiber arrays can generate capillary pressure and drive the infusion of Li into Ni foam within 30 s at 350 °C. Other nanoarrays, such as ZnO nanorods,^[^ ^59^ ^]^ carbon‐nitride nanowires,^[^ ^60^ ^]^ and TiC/C core–shell arrays,^[^ ^61^ ^]^ have been designed to enhance the lithiophilicity of metallic substrates. In addition, 3D porous Cu derived from dealloying Zn from Cu–Zn alloys has been used as a Li host to store molten Li through a thermal infusion method.^[^ ^62^ ^]^ Other 3D Cu‐based current collectors, such as 3D Cu consisting of numerous protuberant tips^[^ ^20^ ^]^ and porous Cu with vertically aligned channels,^[^ ^63^ ^]^ have also shown potential applications as Li hosts. Nevertheless, the practical application of metallic current collectors as hosts may be prevented due to their high densities.

**Figure 4:**
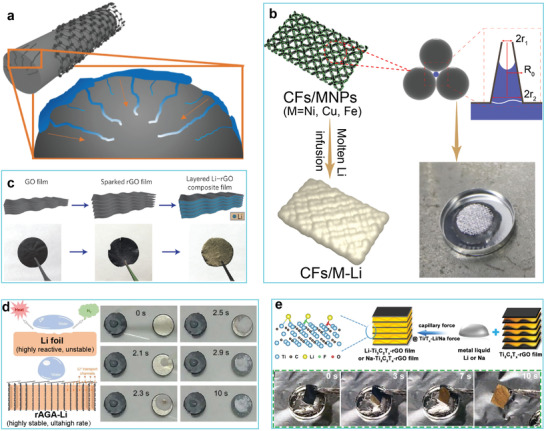
a) Schematic illustration of the infusion process for impregnating the nanocrevasses of the carbon scaffold with Li/Na. Reproduced with permission.^[^ ^65^ ^]^ Copyright 2019, American Chemical Society. b) Schematic illustration of the infusion process for incorporating Li into carbon fibers (CFs) anchored with abundant metal nanoparticles. Reproduced with permission.^[^ ^66^ ^]^ Copyright 2020, Elsevier. c) Schematic representation of the preparation of layered Li–rGO composite film via a sparked reaction between Li and rGO. Reproduced with permission.^[^ ^67^ ^]^ Copyright 2016, Springer Nature. d) Comparison of the stability, safety, and water tolerance of accordion‐like graphene oxide array (rAGA)–Li and Li foil. Reproduced with permission.^[^ ^68^ ^]^ Copyright 2019, Wiley‐VCH. e) Schematic representation of the preparation of Li/Na–Ti_3_C_2_T*_X_*–rGO composite anode and the corresponding wetting process. Reproduced with permission.^[^ ^72^ ^]^ Copyright 2019, American Chemical Society.

Carbon fibers show great potential for energy storage due to their high specific surface area, high mechanical strength, lightweight, and self‐supporting properties.^[^ ^64^ ^]^ They have recently been studied for use in rechargeable LMBs, but their surfaces show a poor affinity for Li, greatly limiting their application as Li hosts. Numerous efforts have been devoted to improving the surface characteristics of carbon fibers. One seminal work for developing abundant nanocrevasses on carbon fibers at 500 °C was conducted by Lee and co‐workers.^[^ ^65^ ^]^ Large capillary forces that arise from the nanocrevasses drove the rapid infusion of molten Li (**Figure** **4**a) into the carbon scaffold. Guided by capillary theory, Guo and co‐workers introduced nonreactive transition metal nanoparticles (e.g., Ni, Cu, and Co) onto carbon cloth surfaces (Figure 4b).^[^ ^66^ ^]^ A deformed capillary‐like structure with conical nanogaps, derived from aggregated transition metal nanoparticles, gaves rise to a Laplace pressure acting on the liquid Li (Figure 4b), allowing it to spread onto the carbon cloth at 220 °C. The thickness of composite Li anodes based on commercial carbon cloth usually reaches hundreds of micrometers, however, which may lead to low energy density for batteries assembled from this material. Therefore, hosts with a thickness of <50 µm have been proposed. Cui and co‐workers constructed an ultrathin (≈50 µm) composite Li anode through a spark reaction between Li and a layered reduced graphene oxide (rGO) film (Figure 4c).^[^ ^67^ ^]^ The capillary force generated from the uniform nanogaps between rGO sheets, together with the strong binding of the lithiophilic functional groups in the rGO, enabled quick infusion of molten Li into the matrix. Then, a 3D accordion‐like graphene oxide array (rAGA) host with vertically aligned graphene sheets on the bottom and slope‐aligned graphene sheets on top was explored as a Li host (rAGA‐Li) (Figure 4d).^[^ ^68^ ^]^ Due to the porous structure from the vertically aligned graphene sheets and capillary forces, the bottom layer was gradually wetted by molten Li. Moreover, the slope‐aligned sheets on the roof layer exhibited great hydrophobicity, which was designed to prevent water‐erosion of the underlying Li metal to ensure safety (Figure 4d). Transition‐metal carbides and carbonitrides (MXenes) are graphene‐like 2D materials that have been developed as Li hosts due to their unique lithiophilic surfaces.^[^ ^69^, ^70^, ^71^ ^]^ For instance, Cao and co‐workers^[^ ^72^ ^]^ fabricated a freestanding porous Ti_3_C_2_T*_X_*‐rGO membrane which formed a micro/nanostructure once it came into contact with molten Li (Figure 4e). The capillary pressure from the micro/nanogaps and the binding between the lithiophilic Ti_3_C_2_T*_X_* surface and the Li both served as driving forces for the rapid infusion of molten Li (≈10 s) (Figure 4e). Carbon nanotube (CNT) microspheres with abundant inner pores prepared by spray‐drying have been applied as Li hosts.^[^ ^73^ ^]^ Acetylene black (AB) was introduced into the carbon nanotube microspheres to lower the Li nucleation barrier of AB.^[^ ^74^ ^]^ Compared with pure CNT microspheres, the CNT‐AB microspheres formed a denser structure after melt infiltration.

Overall, tuning the surface microstructure of substrates has been proven effective for improving the wettability. In this context, exploring advanced surface treatment technologies is the key to producing micro/nanochannels. At present, surface modification technologies include laser microprocessing, laser ablation, magnetron sputtering, and high temperature treatment, which are complicated, time‐consuming, and costly. In addition, the wettability mechanism is normally attributed to the capillary effect of micro/nanochannels. Further endeavors should be devoted to understanding the underlying mechanism, which is beneficial for the exploration of superlithiophilic substrates.

#### 3.2.2 Surface Composition

**Figure 5:**
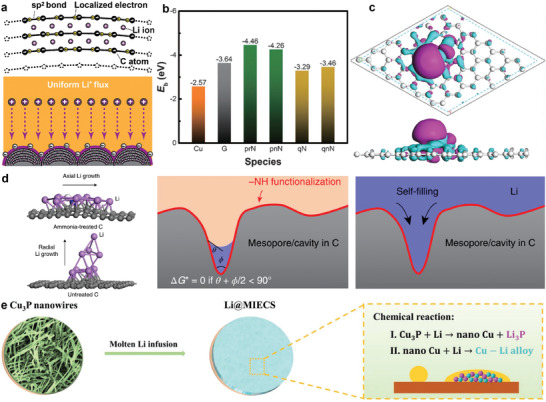
a) Schematic illustrating of the improved Li plating/stripping performance of the Li‐intercalated curved graphite sheet. Reproduced with permission.^[^ ^75^ ^]^ Copyright 2017, American Chemical Society. b) Comparison of binding energy of a Li atom with Cu, graphene (G), and various functional groups of N‐doped graphene. Reproduced with permission.^[^ ^77^ ^]^ Copyright 2017, Wiley‐VCH. c) The differential charge density of N–P‐codoped graphene sheet. Reproduced with permission.^[^ ^78^ ^]^ Copyright 2019, Wiley‐VCH. d) DFT calculation of Li growth on ammonia‐treated and untreated mesoporous carbon surfaces. Reproduced with permission.^[^ ^79^ ^]^ Copyright 2019, Springer Nature. e) Schematic representation of the preparation of Li@MIECS composite anode. Reproduced with permission.^[^ ^81^ ^]^ Copyright 2020, Wiley‐VCH.

The Li wettability of carbon‐based materials can be improved via tuning their intrinsic surface features. Guo and co‐workers reported a Li‐predeposited C host with nanostructures consisting of electronegative curved graphite sheets arranged in a spherical secondary architecture (**Figure** **5**a).^[^ ^75^ ^]^ The hybrid storage material, combining Li intercalation/nanoplating, enhanced the surface electronegativity of C and ensured strong binding between C and Li^+^. Wu and co‐workers^[^ ^76^ ^]^ prepared carbon nanofibers with a high degree of surface graphitization through electrospinning, followed by carbonization at 1200 °C. The carbon nanofibers exhibited significantly improved Li wettability because of the enhanced binding between Li and highly graphitized carbon. Elemental doping is also feasible for enhancing the electronegativity of C, which improves Li^+^ absorption. Zhang and co‐workers^[^ ^77^ ^]^ reported that N‐doped graphene showed a higher binding energy with Li, which was used to guide the nucleation and growth of metallic Li (Figure 5b). Hong et al. reported that electron‐rich elements (e.g., nitrogen, oxygen, and phosphorus) doped with carbon exhibited improved lithiophilicity and wettability. Zhang and co‐workers^[^ ^78^ ^]^ carried out density functional theory (DFT) calculations to show that N and P codoping in a carbon scaffold greatly improved the binding energy between Li atoms and the doped graphene sheets and improved the wettability of carbon fibers (Figure 5c). The whole melting process required only 4 s at 315 °C. Liu and co‐workers^[^ ^79^ ^]^ designed amine‐functionalized 3D mesoporous carbon fibers as Li host. DFT calculation verified that the introduced —NH functional groups have strong chemical binding with Li (Figure 5d), which not only endowed the super wettability of the fibers but also navigated the axial growth of Li deposits along the carbon fibers during cycling. Additionally, they further proved that the mesopores in the carbon fibers served as the preferred nucleation sites, which can lead to the well accommodation of Li into the pores, demonstrating a self‐smoothing anode (Figure 5d). Later, parallelly aligned MXene (Ti_3_C_2_T*_x_*) layers have been utilized to guide the horizontal growth of lithium on the MXene layers.^[^ ^71^ ^]^ Carbon nanotubes host featuring lithiophilic‐lithiophobic gradient properties has been designed to direct dense Li deposition from the bottom.^[^ ^80^ ^]^ To keep battery working in the presence of Li dendrites, Yang's group fabricated a polyimide (PI)‐clad copper grid current collector (E‐Cu) via photolithographic‐level conformity technology to laterally direct the encapsulation of Li metal within the interior Cu scaffold.^[^ ^29^ ^]^ Recently, Wen and co‐workers reported that partial phosphorylation of 3D Cu to generate Cu_3_P nanowires could be used to transform a substrate from lithiophobic to lithiophilic via the reaction between Cu_3_P and molten Li to generate a mixed ion/electron‐conducting skeleton (MIECS) (Figure 5e).^[^ ^81^ ^]^ Moreover, the discharge products of Cu–Li alloys act as both an electron‐conducting skeleton and Li nucleation sites. Li_3_P serves as the ion‐conducting skeleton and ensures the structural stability of the thus‐formed 3D Li anodes. This ion/electron conductive network in situ formed during molten Li infusion offers a new concept for designing robust composite anodes.

In conclusion, Li wettability is highly dependent on the surface composition of substrates. Artificially introducing functional groups or doping various elementals into the substrates have progressed and are now mainstream approaches. First‐principles calculation has been developed as the most powerful means to screen elements or functional groups that have strong binding with Li. Substrates with intrinsic functional sites (e.g., MXenes) will attract widespread attention. Bio‐based polymeric materials, which always possess abundant characteristic elements, show great potential for preparing low‐cost carbon materials featuring functional elements.

#### 3.2.3 Surface Coating

As stated earlier, substances that can react with Li to form alloys or compounds may lower the interior atomic binding force of Li and thus decrease the surface tension of liquid Li and facilitate its wettability. Therefore, reactive coatings have been developed to tune the Li wettability of materials. Compared with regulating the intrinsic characteristics of Li droplets, the regulation of substrates has several advantages: i) a relatively mild temperature ensures operational safety and low energy consumption; ii) only trace element additives are needed to satisfy these requirements; and iii) it is suitable for many substrates. According to their negative value of Δ*G*, many metallic oxides, including ZnO,^[^ ^82^ ^]^ SnO_2_,^[^ ^83^, ^84^ ^]^ MnO_2_,^[^ ^85^ ^]^ Co_3_O_4_,^[^ ^86^, ^87^ ^]^ V_2_O_5_,^[^ ^88^ ^]^ and Cu*_x_*O,^[^ ^89^, ^90^, ^91^ ^]^ can be utilized as coatings to regulate the wettability of substrates toward liquid Li. For example, Hu and co‐workers^[^ ^83^ ^]^ coated SnO_2_ on the surfaces of carbon fibers through a solution immersion method. SnO_2_ reacted with molten Li to form Li_22_Sn_5_ alloy during the melting process, which facilitated the rapid infusion of Li, which only required 27 s. Lu and co‐workers modified carbon sheet with Co_3_O_4_ nanofibers, and reported that the Co_3_O_4_ nanofibers promoted the infusion of molten Li at 200 °C within 15 s.^[^ ^86^ ^]^ The discharge products of Co/Li_2_O nanofibers were proven to accommodate the deposited Li and redistribute the Li^+^ flux. Zhou and co‐workers reported that Cu*_x_*O‐treated carbon fibers led to a complete spreading of liquid Li in only 5 s at 320 °C.^[^ ^90^ ^]^

In the case of the metallic oxides, the improved wettability is due to the following two reactions

(4) $$\begin{array}{c} 2y\mathrm{Li}+M_{x}O_{y}=xM+yLi_{2}O \end{array}$$

(5) $$\begin{array}{c} \mathrm{Li}+M=\mathrm{LiM} \end{array}$$

**Figure 6:**
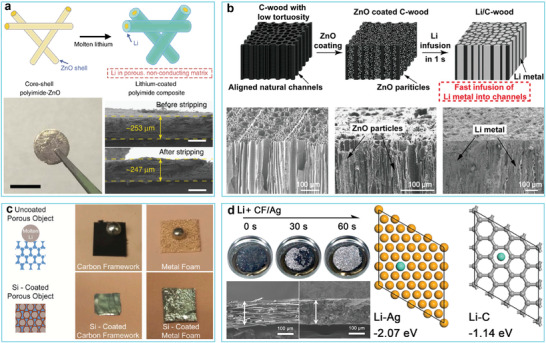
a) Schematic diagram of the fabrication of the Li‐coated polyimide (PI) substrate. Reproduced with permission.^[^ ^82^ ^]^ Copyright 2016, Springer Nature. b) Schematic illustration of the infusion process for infusing Li into the carbonized wood. Reproduced with permission.^[^ ^93^ ^]^ Copyright 2017, National Academy of Sciences. c) Comparison of the preparation of Li anode composites with and without Si coating. Reproduced with permission.^[^ ^34^ ^]^ Copyright 2016, National Academy of Sciences. d) Preparation of layered Li–CF/Ag composite and its corresponding structural characterization. Reproduced with permission.^[^ ^105^ ^]^ Copyright 2018, Elsevier.

The thus‐formed metal nanoparticles or alloys can guide Li nucleation and growth, enabling long‐term cycling stability for composite Li anodes. ZnO has received great attention due to its following advantages: 1) the spontaneous reaction between ZnO and molten Li provides the driving force for wettability;^[^ ^82^, ^92^ ^]^ 2) it shows good adhesion to various substrates, including metal foams, polymer fibers, carbon‐based materials, and porous garnet solid‐state electrolytes;^[^ ^35^, ^82^, ^93^ ^]^ 3) even at nanoscale thicknesses, ZnO coatings still display superior wetting behavior;^[^ ^44^ ^]^ 4) the in situ formation of Li–Zn alloys with a higher diffusion coefficient than bulk metal Li facilitates a rapid reaction and stable Li electrodeposition.^[^ ^5^ ^]^ One seminal work involving the regulation of the wettability of Li via the ZnO layer was conducted by Cui and co‐workers.^[^ ^82^ ^]^ A polyimide (PI) scaffold decorated with a thin 30 nm ZnO coating demonstrated superior adsorption ability toward liquid Li. The Li composite anode suppressed dendrite growth and maintained a stable electrode volume change (**Figure** **6**a). Carbonized wood has aroused great attention due to its well‐aligned channels and high porosity, and has been developed as a potential Li host (Figure 6b).^[^ ^93^ ^]^ Molten Li can be easily absorbed into the channels of wood to form a composite Li/C anode assisted by a nanoscale ZnO layer. Due to the confinement in channels, the Li was well entrapped in the framework without obvious volume variation (Figure 6b). These groundbreaking works were followed by enormous worldwide research, in which various current collectors, including carbonized eggplant,^[^ ^94^ ^]^ carbon fibers,^[^ ^95^, ^96^ ^]^ 3D graphene,^[^ ^97^, ^98^ ^]^ copper foam,^[^ ^99^ ^]^ and nickel foam,^[^ ^59^, ^100^ ^]^ were developed as efficient hosts. It is also believed that ZnO nanoarrays deposited on current collectors can generate capillary forces that promote wetting.^[^ ^59^ ^]^ Despite these great achievements, the Li_2_O formed in situ from metal oxide coatings may prevent substrates from being wetted by Li due to the high formation energy (0.23 J cm^−2^) of the Li_2_O/Li interfaces. Li and co‐workers verified this by comparing two Ni foam substrates with or without prelithiated ZnO before wetting.^[^ ^100^ ^]^ Later, Cui and co‐workers used in situ imaging to observe that Li_2_O films suppressed the Li wettability.^[^ ^44^ ^]^

Pure nonmetallic or metallic substances that react with molten Li to generate Li‐rich alloys free from Li_2_O, such as Si,^[^ ^34^, ^101^, ^102^, ^103^ ^]^ Sn,^[^ ^104^ ^]^ and Ag,^[^ ^105^ ^]^ are preferred. For instance, Cui and co‐workers^[^ ^34^ ^]^ prepared a stable lithium–scaffold composite electrode by infusing molten Li into silicon (Si)‐modified carbon fibers (Figure 6c). A binary lithium silicide alloy formed through the reaction between Si and molten Li acted as the “lithiophilic” site, which was beneficial to the ultrafast infusion of Li in only 9 s at 300 °C (Figure 6c). Zheng and co‐workers^[^ ^102^ ^]^ fabricated a lithiophilic host by growing Si nanowire arrays on commercial carbon cloth. The Si nanowires improved the wettability of the C skeleton on liquefied Li. The as‐obtained Li/C anode demonstrated low volume changes because of the confinement of the C scaffold. Inspired by these groundbreaking works, Ag‐coated carbon fibers have also been developed as substrates for molten Li.^[^ ^105^ ^]^ Ag particles react with Li to form Li–Ag alloys that show strong binding with Li (Figure 6d). In addition, the Ag particles can also serve as the active sites and regulate Li nucleation and growth during Li plating. Hou and co‐workers encapsulated Sn nanoparticles into mesoporous CMK‐3 to obtain a CMK‐3/Sn composite that reacted with molten Li at high temperatures to form Li*_x_*C and Li*_y_*Sn alloys that promoted uniform Li deposition.^[^ ^104^ ^]^ Other elements, such as Al,^[^ ^106^ ^]^ Ge,^[^ ^107^ ^]^ Mg,^[^ ^108^ ^]^ and Au,^[^ ^109^ ^]^ have also been applied as surface coatings to improve Li wettability.

In addition to the intrinsic characteristics of substances, the coating thickness should also be taken into consideration.^[^ ^44^ ^]^ The critical thickness (defined as the thickness of the Li/substrate at a contact angle of 45°) is highly dependent on the Gibbs free energy and the amount of Li_2_O formed in situ. Compared with Al_2_O_3_ and TiO_2_, ZnO shows a lower critical wetting thickness due to a more negative Gibbs free energy (−20.0 × *t* × 10^9^ J cm^−2^ (ZnO), −8.5 × *t* × 10^9^ J cm^−2^ (TiO_2_), −9.2 × *t* × 10^9^ J cm^−2^ (Al_2_O_3_), where *t* is the coating thickness) and less Li_2_O is formed during the reaction. Due to the deleterious effects of the Li_2_O formed in situ, the critical wetting thickness of metallic oxides is always higher than those of the corresponding pure metals. For example, the critical wetting thickness of Al_2_O_3_ is >600 nm, while that of pure Al metal is about 40 nm. Moreover, the contact angle is strongly correlated with the thickness of coatings, where increasing the coating thickness leads to a gradual decrease in the contact angle and thus improved wettability. Despite the great progress that has occurred in the regulation of nucleation and wetting, the use of surface coatings is still restricted by their poor structural stability, which is due to their serious volume expansion/contraction during continuous alloying/dealloying processes. Therefore, the intimate interaction between the modified layers and matrices should be taken into consideration for future development of LMBs toward practical applications.

## 4 Li Composite Anodes in Solid‐State Batteries

**Figure 7:**
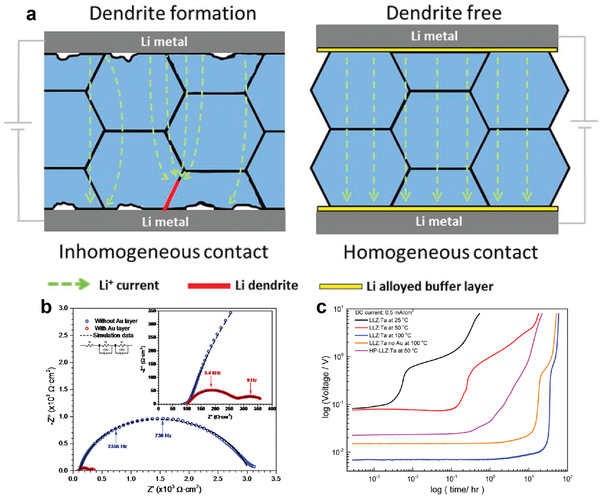
a) Schematic illustration of the interface improvement from using a Li alloyed buffer layer. b) Impedance spectra and c) constant direct current measurements on Li/SSE/Li symmetrical cells with and without Au buffer layers. Reproduced with permission.^[^ ^124^ ^]^ Copyright 2016, American Chemical Society.

Solid‐state electrolytes (SSEs) show a wider stability window (0–5 V) in batteries than organic liquid electrolytes. They also show good thermal stability, high mechanical rigidity, and nonflammability.^[^ ^110^, ^111^, ^112^ ^]^ Common SSEs include polymer electrolytes,^[^ ^113^, ^114^, ^115^ ^]^ polymer–inorganic composite electrolytes,^[^ ^116^, ^117^, ^118^ ^]^ and inorganic electrolytes.^[^ ^119^, ^120^, ^121^ ^]^ Amongst these, inorganic electrolytes have received great attention due to their high ionic conductivity and extremely high Young's modulus, which can enhance the safety of Li–metal batteries. Garnet‐type SSEs, such as Li_7_La_3_Zr_2_O_12_ (LLZO), have been widely explored because of their high chemical inertness toward Li metal.^[^ ^122^, ^123^ ^]^ When an SSE is matched with a Li metal anode, however, there is poor solid‐solid contact between Li and the SSE (**Figure** **7**a), which results in large interfacial impedance, uncontrollable Li dendrite growth, rapidly decaying performance, and a short lifespan.^[^ ^124^ ^]^ For instance, Li/LLZO/Li cells displayed a high total surface area resistance of 3000 Ω cm^2^, a value that is ten times higher than for their counterparts with an Au buffer layer (Figure 7b), which can be run for 22.5 h at 0.5 mA cm^−2^ without forming a short circuit (Figure 7c).^[^ ^124^ ^]^ In order to target the poor interface contact, many efforts have been devoted to applying mechanical pressure to maximize the SSE–Li physical contact,^[^ ^125^, ^126^ ^]^ using polymer interlayers to adhere to Li and the SSE,^[^ ^127^, ^128^, ^129^ ^]^ controlling the garnet composition to improve its wettability by Li,^[^ ^130^ ^]^ and utilizing thermal infusion strategies to construct integral SSE–Li anodes.^[^ ^131^ ^]^ Compared with the other strategies, thermal infusion is considered to be the most effective way to minimize interfacial resistance.

Directly spreading molten Li on an SSE surface to obtain smooth Li anode/SSE interfaces is effective, yet challenging. SSEs commonly exhibit lithiophobicity because their surfaces are inevitably attacked by moisture and covered by Li_2_CO_3_, LiOH, and Li_2_O.^[^ ^132^, ^133^, ^134^ ^]^ Thermal treatment,^[^ ^135^, ^136^ ^]^ surface chemical modification,^[^ ^137^, ^138^ ^]^ and polishing methods^[^ ^139^ ^]^ are employed to remove the impurities from the SSE surface, and thus improve their wettability toward liquid Li. In this context, Goodenough and co‐workers^[^ ^136^ ^]^ proposed a facile method to remove these impurities via a reaction between garnet and C at 700 °C. The C‐treated garnet was wetted by molten Li. Electrochemical impedance spectroscopy (EIS) at the Li/garnet interface decreased from 1210 (pristine garnet) to 28 Ω cm^2^ (C‐treated garnet) at 25 °C. Guo and co‐workers^[^ ^138^ ^]^ dropped an ammonium fluoride solution on the Li_6.4_La_3_Zr_1.4_Ta_0.6_O_12_ (LLZTO) surface and found that the surficial contaminants could be converted into fluorinated interphase at a moderate temperature (<180 °C). The fluorinated interface formed in situ not only drove the wetting process but also guaranteed the air stability of the solid electrolyte. Recently, Duan and co‐workers carried out DFT calculations to show that Li_2_O, not Li_2_CO_3_, might determine the lithiophobicity of garnet surfaces.^[^ ^139^ ^]^ They removed impurities from the garnet pellets and found that the EIS value of the charge transfer resistance for Li|LLZT|Li symmetric cells was only 6.95 Ω cm^2^, which was remarkably lower than for the cells with untreated Li/LLZT interfaces (492.6 Ω cm^2^). Likewise, the polished interface exhibited a greatly improved ability to suppress Li dendrite growth, as evidenced by its high critical current density (13.3 mA cm^−2^). Despite great achievements in reducing the interfacial impedance using these approaches, continuous endeavors should be devoted to exploiting simple and universal treatment techniques for the improved wettability of SSEs for solid‐state Li batteries.

**Figure 8:**
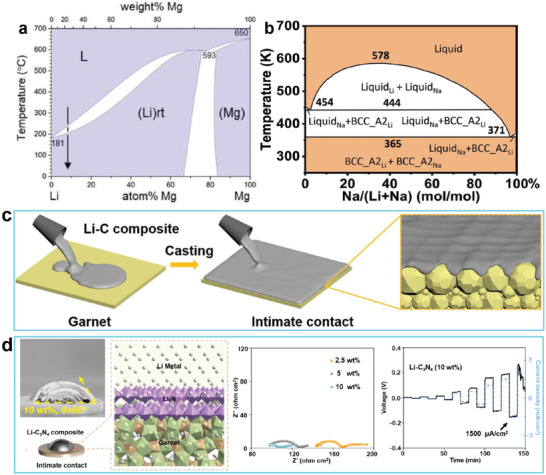
a) Phase diagram of Li–Mg alloy. Reproduced with permission.^[^ ^49^ ^]^ Copyright 2019, Wiley‐VCH. b) Phase diagram of Li–Na alloy. Reproduced with permission.^[^ ^140^ ^]^ Copyright 2020, American Chemical Society. c) Schematic illustration of casting Li–C composite on garnet SSEs. Reproduced with permission.^[^ ^50^ ^]^ Copyright 2019, Wiley‐VCH. d) Schematic representation of the fabrication of Li–C_3_N_4_ composite and the interfacial contact of Li–C_3_N_4_/garnet. Reproduced with permission.^[^ ^51^ ^]^ Copyright 2020, Wiley‐VCH.

Adding different elements to molten Li to decrease the surface tension of droplets and improve the wettability of Li toward SSEs has proven feasible for enhancing the electrochemical performance of solid‐state batteries. Due to its relatively low density (1.7 g cm^−3^), Mg does not significantly degrade the energy density of cells and has been used as a dopant. Hu and co‐workers reported that Mg was miscible with Li and formed a Li–Mg binary alloy at a moderately high temperature (350 °C) (**Figure** **8**a).^[^ ^49^ ^]^ Thus, they proposed introducing 20 wt% Mg into liquid Li to improve the affinity between Li and the garnet pellets, and to smooth the contact between the Li anode and the SSE. Later, Li and co‐workers^[^ ^140^ ^]^ found that doping Li with a trace amount of metallic Na generated a Li–Na intermediate phase that induced subsequent Li spreading over the SSE surface (Figure 8b). In addition to metals, nonmetal dopants are also used. Recent studies indicated that lithium–graphite (Li–C) composites showed lower fluidity and higher viscosity than pure Li, which enabled them to firmly adhere to garnet. A composite with 50 wt% C was doped into metallic Li to improve its wettability on garnet‐type Li_6.5_La_3_Zr_1.5_Ta_0.5_O_12_ (LLZTO) pellets at 250 °C, enabling it to realize tight conformal contact between Li and LLZTO (Figure 8c).^[^ ^50^ ^]^ Later, Li–C_3_N_4_ composites (10 wt% C_3_N_4_) were investigated for a similar purpose (Figure 8d). Due to the improved wettability, Li was bound tightly with the SSE, leading to a greatly decreased interfacial resistance (11 Ω cm^2^).^[^ ^51^ ^]^ The ionic conductor–Li_3_N layer formed in situ by reacting Li with C_3_N_4_ at the interface enabled rapid ion transport, contributing to an improved critical current density (1.5 mA cm^−2^) (Figure 8d).

**Figure 9:**
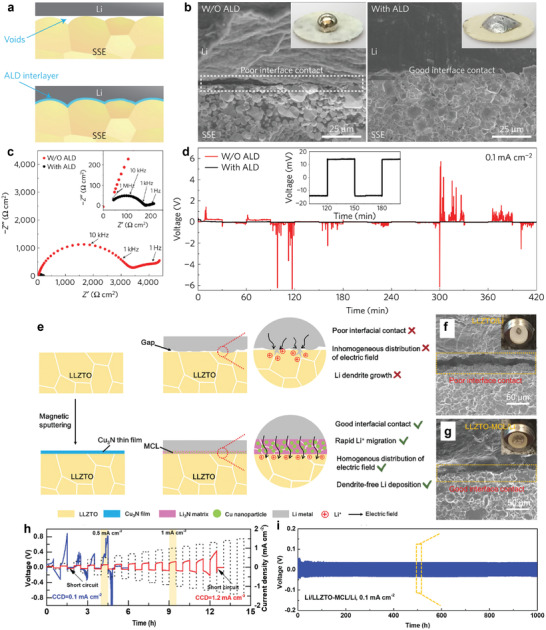
a) Schematic illustrations of the Li/SSEs interface. b) SEM images of the Li/SSEs interface. c) EIS profiles of the Li|LLCZN|Li symmetric cells with or without Al_2_O_3_ coating. d) Comparison of long‐term cycling stability of the Li|LLCZN|Li symmetric cells with or without an Al_2_O_3_ coating. Reproduced with permission.^[^ ^141^ ^]^ Copyright 2016, Springer Nature. e) Schematic diagram of the mixed conductive layer (MCL) protected Li/LLZTO interface. SEM images of Li/LLZTO interface (optical images in insets) f) with and g) without MCL. h) Critical current density (CCD) of the Li/LLZTO/Li and the Li/LLZTO‐MCL/Li cells. i) Comparison of long‐term cycling stability of the Li/LLZTO/Li and the Li/LLZTO‐MCL/Li cells. Reproduced with permission.^[146]^ Copyright 2020, The Royal Society of Chemistry.

Introducing a coating layer on an SSE surface has been developed as another strategy for improving Li wettability. To this end, various ultrathin coatings, such as ZnO,^[^ ^35^ ^]^ Al_2_O_3_,^[^ ^141^ ^]^ C,^[^ ^142^ ^]^ Si,^[^ ^143^ ^]^ Al,^[^ ^144^ ^]^ Ge,^[^ ^107^ ^]^ Ag,^[^ ^145^ ^]^ and Mg^[^ ^108^ ^]^ have been employed on SSE surfaces to transform them from lithiophobic to lithiophilic. Recently, Hu and co‐workers^[^ ^141^ ^]^ demonstrated that introducing a nanoscale Al_2_O_3_ coating on garnet‐like Li_7_La_2.75_Ca_0.25_Zr_1.75_Nb_0.25_O_12_ (LLCZN) provided perfect wettability with Li and intimate surface contact without interfacial voids (**Figure** **9**a). Without a coating, large microscopic gaps at the interface were detected (Figure 9b). The poor interfacial contact resulted in a high interfacial impedance of 1710 Ω cm^2^, which was significantly decreased to 1 Ω cm^2^ by the use of a coating (Figure 9c). Due to the conformal garnet/Li interface, a Li|LLCZN|Li symmetric cell was able to cycle stably for 420 h with a low voltage polarization of 13 mV at 0.1 mA cm^–2^ (Figure 9d). Later, they plated an ultrathin layer of amorphous Si on the SSE surface and found that the spontaneous reaction between Si, Li, and the products of lithiated Si changed the SSE from superlithiophobic to superlithiophilic.^[^ ^143^ ^]^ The Li wetting rate of the Si‐treated SSEs was only 4 s near 200 °C. The interfacial resistance between Li and the Si‐coated SSEs was nearly seven times lower than that for Li/bare SSEs. Unlike the Si interface layer, which reacted with Li to remain just at the Li/SSEs interface, the Mg layer dissolved into the molten Li and diffused into the bulk Li metal, leading to continuous and intimate interfacial contact between Li and the SSE.^[^ ^108^ ^]^ Yang and co‐workers^[^ ^142^ ^]^ recently used a pencil to draw a soft graphite‐based layer on an SSE surface. The reaction between molten Li and the graphite layer drove the wetting process, and the in situ formation of a LiC*_x_* interface layer significantly decreased the interfacial impedance.

The growth of Li dendrites is likely to be arrested to some extent when current densities are as low as 1.0 mA cm^−2^. Once the current densities exceed 1.0 mA cm^−2^, however, Li dendrites may grow along the grain boundaries of SSEs. Therefore, Sun and co‐workers^[^ ^146^ ^]^ developed a Cu_3_N coating to improve interfacial contact as well as arresting Li propagation (Figure 9e). The spontaneous reaction between Cu_3_N and molten Li provided a driving force for wetting and smoothing the Li/Li_7_La_3_Zr_2_O_12_ interface contact (Figure 9f,g). The reaction product, Li_3_N, had a high Li^+^ ion conductivity (10^−3^ S cm^−1^ for Li_3_N at room temperature), and the well‐dispersed Cu nanoparticles enabled a uniform Li^+^ distribution and homogeneous Li deposition. Due to these advantages, a high critical current density of 1.2 mA cm^−2^ and stable cycling for over 1000 h were achieved in a Li_3_N/Cu‐protected Li symmetrical cell (Figure 9h,i). Following this principle, a SnN*_x_* nanolayer that can react with molten Li to in situ generate Li−Sn alloy and Li_3_N was introduced on the surface of SSEs to improve the electrochemical performance of the assembled battery.^[^ ^147^ ^]^

**Figure 10:**
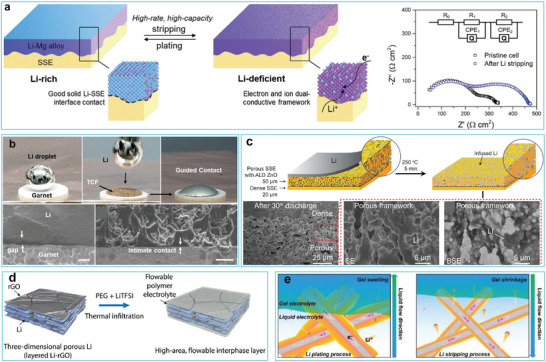
a) Li plating/stripping behavior within the Li–Mg alloy framework. Reproduced with permission.^[^ ^49^ ^]^ Copyright 2019, Wiley‐VCH. b) Optical observation and SEM images of the wettability of molten Li on garnet with and without TCF. Reproduced with permission.^[^ ^149^ ^]^ Copyright 2020, Wiley‐VCH. c) Schematic illustration of the fabrication of a porous dense bilayer as an integral Li/EES configuration. Reproduced with permission.^[^ ^131^ ^]^ Copyright 2018, Elsevier. d) Schematic illustration of 3D Li–rGO composite anode filled with liquid‐like PEG‐LiTFSI. Reproduced with permission.^[^ ^152^ ^]^ Copyright 2017, American Association for the Advancement of Science. e) Schematic illustration of Li plating/stripping behavior on 3D Li/CF composite anode integrated with trimethylolpropane triglycidyl ether (TTE) gel electrolyte. Reproduced with permission.^[^ ^153^ ^]^ Copyright 2020, Wiley‐VCH.

Because of the hostless nature of Li, Li anodes experience large volume expansion/contraction over prolonged cycling, which may result in interfacial fluctuations and possible detachment of the Li anode from the SSE.^[^ ^148^ ^]^ Therefore, solely improving the wettability is not enough to address safety concerns and rapid performance decay. Recently, Hu and co‐workers showed that the substitution of a Li‐rich alloy for Li foil can ensure stable solid Li/SSE interfacial contact and withstand Li anode volume changes, as evidenced by the small EIS differences before and after Li exhaustion from a Li–Mg alloy (**Figure** **10**a).^[^ ^49^ ^]^ Placing a lithiophilic 3D host on an SSE surface can induce the infiltration of Li inside the scaffold and blur the Li/SSE interface (Figure 10b).^[^ ^149^ ^]^ Without the treated copper foam (TCF) as the lithiophobic host, a large physical gap was observed between the Li metal and the garnet. By contrast, when using a TCF, molten Li was rapidly infused into the TCF and formed an intimate contact with the garnet (Figure 10b). Recent research has verified that hybrid Li anodes with Li infused into a porous and highly Li^+^ conductive framework may be the most ideal approach to address the above issues.^[^ ^150^, ^151^ ^]^ In this context, Hu and co‐workers engineered a porous, dense bilayer as an integral EES configuration (Figure 10c).^[^ ^131^ ^]^ The bottom porous garnet SSE decorated with an ultrathin ZnO nanolayer enabled accommodation of the molten Li inside to generate continuous ion and electron pathways. The dense upper garnet SSE inhibited the formation of Li dendrites and also remained in contact with the bottom Li composite due to the similar characteristics of the two layers. As a result, a volumetrically stable Li metal composite anode with good interfacial contact was constructed (Figure 10c).

Due to the sluggish Li^+^ transfer through the bulk electrolyte and electrode–electrolyte interface, and the limited solid‐solid contact surface, the assembled batteries usually exhibit large polarization, rapidly deteriorating performance, and a short lifespan.^[^ ^154^, ^155^ ^]^ These problems can be addressed by encapsulating trace amounts of liquid or gel electrolytes into these systems. Fluid electrolytes provide continuous ionic contact and fast ion‐conductive pathways that ensure uniform Li deposition and the high‐power operation of cells. A seminal work for incorporating poly(ethylene glycol) plasticized by bis(trifluoromethane)sulfonimide Li salt into a layered rGO host (Li‐rGO) was reported by Cui and co‐workers (Figure 10d).^[^ ^152^ ^]^ Plasticized polyethylene glycol (PEG) with a low molecular weight served as a viscous semiliquid and enlarged the electrode–electrolyte contact area, while the rGO scaffold ensured a constant Li anode volume. Later, Guo and co‐workers^[^ ^153^ ^]^ designed a 3D Li/carbon fiber (CF) anode by infusing Li into fluorine (F)‐doped carbon fibers, which was followed by the in situ polymerization of a liquid electrolyte inside the Li anode (Figure 10e). The Li intake rate must be strictly controlled because a balance between the Li metal and the electrolyte is critical for rapid transportation dynamics and battery performance.

In conclusion, reducing the interfacial resistance and slowing the Li dendrite growth via the thermal infusion method have evolved into the two key points for the exploration of solid‐state composite Li metal anodes. Nevertheless, how to keep the Li volume stable and achieve fast Li^+^ transport inside the anode remain critical but have not yet been fully explored. Integral Li anodes with porous SSEs in combination with Li anodes or 3D Li anodes in conjunction with SSEs are perceived as the ideal anode configurations to construct electron/ion dual networks. By importing some flowing liquid/gel electrolytes into the integral anodes, the solid‐state batteries can be endowed with fast ion transport, good anode/SSE contact, uniform Li deposition, and finally, superior cycling stability.

## 5 Summary and Outlook

The rapid development of rechargeable Li metal batteries has resulted in the exploration of Li metal anodes with high safety and long cycling lifetimes. The encapsulation of Li into substrates via thermal infusion is considered to be the most feasible process for the large‐scale fabrication of composite Li anodes. Although only short‐term studies have been conducted, numerous stable composite Li anodes have been constructed using melt infusion methods that have matched the performance of state‐of‐the‐art cathodes. Thus, we have reviewed recent advances in Li wetting on lithiophilic substrates and the corresponding mechanisms. Surface tension and capillary pressure were two intrinsic factors governing the wetting efficiency. Elevating the temperature and heteroatomic doping are two feasible methods for improving Li wettability by weakening the effects from neighboring Li atoms and decreasing the surface tension of the liquid Li. Devising micro/nanostructures on substrate surfaces can generate capillary pressure that drives the rapid infiltration of Li.

Increasing the heating temperature is convenient and straightforward, but it shows high substrate selectivity, and relatively high temperatures are needed for superlithiophobic hosts. Regulating Li droplet characteristics by introducing elemental additives is effective for decreasing the surface tension of liquid Li, but it only works well at a certain additive content, and excessive additives may impair the overall specific capacity of composite Li anodes. Decorating substrates with nanolayers requires only small amounts of additives but it can realize excellent wettability. Additionally, substrates with surface microstructures can facilitate Li intake through capillary pressure.

Later, we discussed the application of Li composite anodes in liquid electrolytes and SSEs. Due to the significant differences in interfacial contact between the two electrolytes systems, the focus for constructing composite Li anodes is slightly different for the two cases. Liquid systems outperform SSEs in the portable electronics and electric vehicle market because of their mature processing technology and superior performance, and they will continue to dominate in the market for the long term, although the rapid consumption of Li resource has aroused attention to Li utilization. Ultrathin Li metal anodes with a thickness of 20–40 µm can show high Li utilization of 50–100% based on the cathode loading (≥4.0 mA h cm^−2^), the anode to cathode capacity ratio (≤2), and the surface area of the Li anode (Φ10, 0.785 cm^−2^). Due to its intrinsic poor mechanical properties and the high viscosity characteristic of Li, it is believed to be very challenging to prepare ultrathin Li with a thickness <30 µm by rolling technology. Infusing molten Li into the substrates might be the optimal strategy to prepare ultrathin Li, wherein, the substrate selection is most important. Ultrathin, ultrastrong, porous, and flexible 3D substrates with abundant lithiophilic sites are the preferred candidates for liquid systems because they can endow Li anodes with a high energy density and long lifespan.^[^ ^156^ ^]^ High‐active surface area of 3D substrates can induce parasitic reactions between the Li and the electrolyte, contributing to low Coulombic efficiency and low gravimetric energy density caused by the extra injection of liquid electrolyte. In order to realize an electrolyte weight to cathode capacity ratio ≤3 g (Ah)^−1^ and construct a stabilized Li/electrolyte interface, hybrid Li anodes that integrate a framework structure with an artificial interphase have emerged as future anode candidates.^[^ ^33^ ^]^ Encapsulating molten Li into the framework via a thermal infusion method is required to fabricate integral Li anodes. Compared with their liquid counterparts, the exploration of SSE systems is still in its infancy. Due to the rigid solid‐solid contact, good Li/SSE interface contact needs to be ensured in SSE systems. Plating interfacial layers onto SSE surfaces helps to infuse molten Li, while the volume variation of hostless Li presents a challenge to ensuring interfacial stability during prolonged cycling. Designing dense, porous integrated garnet frameworks is a possible direction to take to address the volume variation of the anode,^[^ ^157^ ^]^ although their intrinsic brittleness and high density makes it hard for them to satisfy the demand for flexible solid‐state batteries with high energy density. Devising a ultrathin, flexible, and lithiophilic 3D host with electron/ion conductive pathways is the optimal approach to simultaneously realizing dendrite‐free Li deposition and keeping the Li stable against volume changes, but methods to introduce rapid ion conductive pathways remain challenging.^[^ ^153^ ^]^ To further improve the Li/SSE interface contact and blur interfacial boundaries, depositing a Li^+^ conductive layer on the composite Li anode surface may be used to form a hybrid Li anode. Incorporating trace amounts of flowing liquid/gel electrolytes into the hybrid Li anodes is effective for improving the sluggish Li^+^ transport and addressing the problematic issues for Li anode.

Despite these advances, there are remaining challenges. More feasible methods, such as electric‐field‐induced techniques, are expected to alter the surface tension of molten Li.^[^ ^158^, ^159^ ^]^ From the perspective of choosing a substrate, identifying an ultrathin, low‐density, ion‐conductive 3D host for high‐energy solid‐state batteries is urgent. The Li intake rate into substrates should be strictly controlled to achieve a balance between high Li utilization and superior electrochemical performance. Continuous efforts should be focused on the wetting mechanism and exploring materials to ensure the viability of thermal infusion methods in the near future.

## Conflict of Interest

The authors declare no conflict of interest.
